# A new quadrannulate species of *Orobdella* (Hirudinida, Arhynchobdellida, Orobdellidae) from central Honshu, Japan

**DOI:** 10.3897/zookeys.445.7999

**Published:** 2014-10-13

**Authors:** Takafumi Nakano

**Affiliations:** 1 Department of Zoology, Graduate School of Science, Kyoto University, Kyoto 606-8502, Japan

**Keywords:** Hirudinea, Hirudinida, *Orobdella*, new species, gastroporous, molecular phylogeny, Japan

## Abstract

A new quadrannulate species of *Orobdella*, *Orobdella masaakikuroiwai* **sp. n.**, from the mountainous region of central Honshu, Japan is described. This is only the second small species known within this genus, with a body length of less than 4 cm for mature individuals. Phylogenetic analyses using nuclear 18S rDNA and histone H3 as well as mitochondrial COI, tRNA^Cys^, tRNA^Met^, 12S, tRNA^Val^, 16S, and ND1 markers showed that *Orobdella masaakikuroiwai* **sp. n.** is the sister species of the quadrannulate *Orobdella whitmani* Oka, 1895. Phylogenetic relationships within *Orobdella masaakikuroiwai* **sp. n.** conducted using mitochondrial markers reveled a distinction between eastern and western phylogroups.

## Introduction

The genus *Orobdella* Oka, 1895 is an East Asian terrestrial macrophagous leech taxon assigned to the family Gastrostomobdellidae Richardson, 1971, along with the Southeast Asian terrestrial macrophagous genus *Gastrostomobdella* Moore, 1929 (Richardson 1971). Gastrostomobdellidae was once classified within the suborder Hirudiniformes, which includes jawed blood-feeding taxa (Sawyer 1986). Recent molecular phylogenetic studies revealed that *Orobdella* is part of the suborder Erpobdelliformes, which contains only predaceous leech taxa (Nakano et al. 2012, Oceguera-Figueroa et al. 2011). The monotypic family Orobdellidae Nakano, Ramlah & Hikida, 2012 was established for *Orobdella* based on both morphological differences between *Orobdella* and *Gastrostomobdella* (gastroporal duct of *Orobdella* is tubular and positioned on top of the female organ while in *Gastrostomobdella*, this duct is columnar and vertical in position to the gastropore) and the results of an analysis by Nakano et al. (2012) which rejected the monophyly of these two taxa. Despite their failure to reconstruct the precise phylogenetic relationships of *Orobdella* and *Gastrostomobdella* within Erpobdelliformes, the classification by Nakano et al. (2012) is followed here.

*Orobdella* now consists of 11 nominal leech species from East Asia: nine species known from the Japanese Archipelago (Nakano 2010, 2011b, 2012a, b, c); one present in the Korean Peninsula and adjacent islands (Nakano 2011a, Nakano and Seo 2012, 2014); and the remainder in Taiwan (Nakano and Lai 2012). Species of *Orobdella* are usually large in size, with the body length of mature individuals reaching to around 10 cm (e.g. Nakano (2011b)). The largest species in this genus is the octannulate *Orobdella octonaria* Oka, 1895 recorded from Honshu, Japan, with a body length often greater than 20 cm (Nakano 2012c, Oka 1895). In contrast, the smallest species is the quadrannulate *Orobdella koikei* Nakano, 2012b found in Hokkaido, Japan with a body length of less than 4 cm, but which were considered to be mature due to the presence of developed testisacs. It is also noteworthy that the distribution of *Orobdella koikei* in Hokkaido overlaps with that of the quadrannulate *Orobdella kawakatsuorum* Richardson, 1975, which is present in Hokkaido as well as its adjacent islands and attains a body length of ca. 10 cm (Nakano 2012b, Nakano and Gongalsky 2014).

Several small *Orobdella* leeches were recently collected from east-central Honshu, Japan. Although the bodies of the specimens are up to 3.5 cm in length, some of them already possess an obvious clitellum and they are thus considered to be mature individuals. These leeches are described herein as a new species. The phylogenetic position of this new species was reconstructed using nuclear 18S and histone H3 (H3), and mitochondrial COI, tRNA^Cys^, tRNA^Met^, 12S, tRNA^Val^ and 16S rDNA, and ND1 sequence data.

## Materials and methods

### Sampling and morphological examination

**Figure 1.**
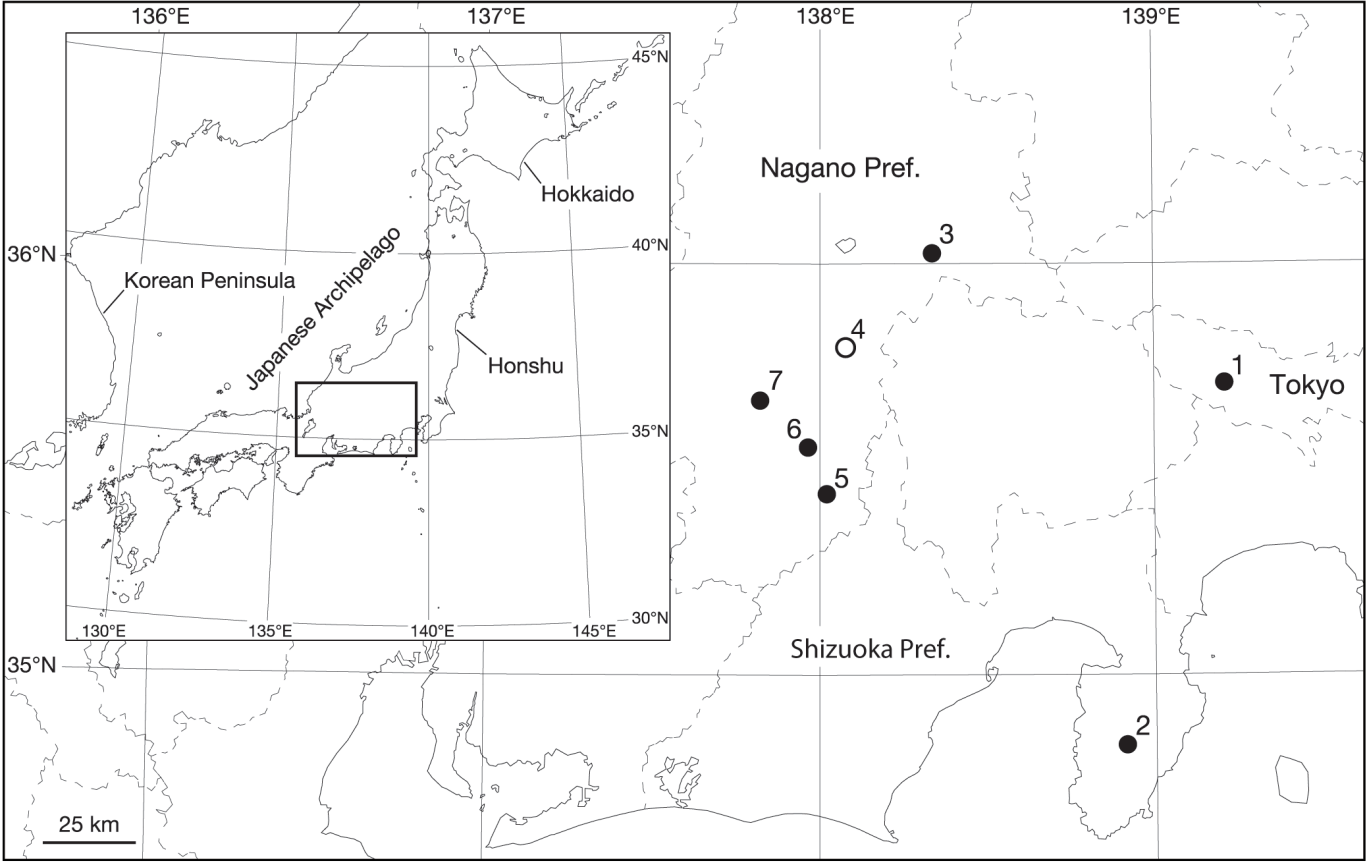
Map showing the collection localities of the specimens examined in this study. Open circle (4) indicates the *Orobdella masaakikuroiwai* sp. n. type locality, and closed circles (1–3, 5–7) indicate additional localities.

Leeches were collected from seven localities in east-central Honshu, Japan (Fig. 1). These seven collection localities are numbered referring to the locality name listed in Table 1. When possible, altitudes above sea level and geographical coordinates for localities were obtained using a Garmin eTrex® GPS unit.

**Table 1.** Collection localities in this study with the information on locality names.

Locality number	Locality name
1	Akiruno, Tokyo Metropolis, Japan
2	Namesawakeikoku Valley, Izu Shizuoka Prefecture, Japan
3	Shibunoyu, Kitayama, Chino, Nagano Prefecture, Japan
4	Mt. Mitsugaisan, Ina, Nagano Prefecture, Japan
5	Shirabisotoge Pass, Ida, Nagano Prefecture, Japan
6	Ikuta, Matsukawa, Nagano Prefecture, Japan
7	Shiojidaira Nature Park, Iizuna, Nagano Prefecture, Japan

The specimens were relaxed by the gradual addition of absolute ethanol to fresh water. For DNA extraction, botryoidal tissue was taken from the posterior part of the body around the caudal sucker of every specimen, and then preserved in absolute ethanol. The rest of the body was fixed in 10% formalin and then preserved in 70% ethanol. Four measurements were taken: body length (BL) from the anterior margin of the oral sucker to the posterior margin of the caudal sucker, maximum body width (BW), caudal sucker length (CL) from the anterior to the posterior margin of the sucker, and caudal sucker width (CW) from the right margin to the left margin of the sucker. Examination, dissection, and drawing of the specimens were accomplished using a stereoscopic microscope with a drawing tube (Leica M125). Specimens used in this study have been deposited in the Zoological Collection of Kyoto University (KUZ).

The numbering convention is based on Moore (1927): body somites are denoted by Roman numerals, and the annuli in each somite are given alphanumeric designations.

### PCR and DNA sequencing

The extraction of genomic DNA from botryoidal tissues preserved in absolute ethanol followed Nakano (2012b). Primer sets for the PCR and cycle sequencing (CS) reactions used in this study were as follows: for 18S, A and L (PCR and CS), C and Y (PCR and CS), and O and B (PCR and CS) (Apakupakul et al. 1999); for histone H3 (H3), H3aF and H3bR (PCR and CS) (Colgan et al. 1998); for COI, LCO 1490 (PCR and CS) and HCO 2198 (CS) (Folmer et al. 1994), and LCO-in (CS) and HCO-out (PCR and CS) (Nakano 2012b); for tRNA^Cys^, tRNA^Met^, 12S, tRNA^Val^, and 16S (tRNA^Cys^–16S), 12SA-out (PCR and CS) and 12SB-in (CS), and 12SA-in (CS) and 12SB-out (PCR and CS) (Nakano 2012b); for tRNA^Leu^ and ND1 (tRNA^Leu^–ND1), LND3000 and HND1932 (PCR and CS) (Light and Siddall 1999). The PCR reaction and DNA sequencing were performed using the modified methods outlined by Nakano (2012a). The 18S, H3 and ND1, and COI and tRNA^Cys^–16S reactions were respectively performed using a GeneAmp PCR System 2700 and a GeneAmp PCR System 9700 (Applied Biosystems). The PCR reaction mixtures were heated to 94 °C for 5 min, followed by 40 cycles at 94 °C (10 s each), 48 °C for 18S, H3, and tRNA^Leu^–ND1 or 45 °C for COI and tRNA^Cys^–16S (20 s), and 72 °C (48 s for 18S, H3 and tRNA^Leu^–ND1 or 1 min 12 s for COI and tRNA^Cys^–16S), and a final extension at 72 °C for 6 min. The sequencing mixtures were heated to 96 °C for 2 min, followed by 40 cycles at 96 °C (10 s each), 50 °C (5 s each), and 60 °C (48 s each). The obtained sequences were edited using DNA BASER (Heracle Biosoft S.R.L.). The DNA sequences listed in Table 2 were newly obtained in this study, and were deposited with the International Nucleotide Sequence Database Collaboration (INSDC).

**Table 2.** Samples used for the phylogenetic analyses. The information on the vouchers is accompanied by the collection locality numbers for *Orobdella masaakikuroiwai* sp. n. (see Fig. 1 and Table 1) and the INSDC accession numbers. Acronym: KUZ, the Zoological Collection of Kyoto University; UNIMAS, the Universiti Malaysia Sarawak.

Species	Voucher	18S	Histone H3	COI	tRNACys–16S	tRNALeu–ND1
*Orobdella masaakikuroiwai* sp. n.	KUZ Z694 Holotype (4)	AB938003	AB938013	AB938006	AB937997	AB938016
*Orobdella masaakikuroiwai* sp. n.	KUZ Z684 (1)			AB938010	AB938001	AB938020
*Orobdella masaakikuroiwai* sp. n.	KUZ Z687 (2)			AB938011	AB938002	AB938021
*Orobdella masaakikuroiwai* sp. n.	KUZ Z689 (5)			AB938005	AB937996	AB938015
*Orobdella masaakikuroiwai* sp. n.	KUZ Z696 (7)			AB938007	AB937998	AB938017
*Orobdella masaakikuroiwai* sp. n.	KUZ Z697 (6)			AB938008	AB937999	AB938018
*Orobdella masaakikuroiwai* sp. n.	KUZ Z699 (3)			AB938009	AB938000	AB938019
*Orobdella dolichopharynx* Nakano, 2011b	KUZ Z120 Holotype	AB663665e	AB698876a	AB679680b	AB679681b	AB828558f
*Orobdella esulcata* Nakano, 2010	KUZ Z29 Holotype	AB663655e	AB698873a	AB679664b	AB679665b	AB828555f
*Orobdella ijimai* Oka, 1895	KUZ Z110 Topotype	AB663659e	AB698877a	AB679672b	AB679673b	AB828559f
*Orobdella kawakatsuorum* Richardson, 1975	KUZ Z167 Topotype	AB663661e	AB698878a	AB679704b	AB679705b	AB828561c
*Orobdella ketagalan* Nakano and Lai, 2012	KUZ Z208 Holotype	AB704785d	AB704786d	AB704787d	AB828582f	AB828563f
*Orobdella koikei* Nakano, 2012b	KUZ Z156 Holotype	AB698883e	AB698882a	AB679688b	AB679689b	AB828560c
*Orobdella mononoke* Nakano, 2012a	KUZ Z224 Holotype	AB698868e	AB698869a	AB698866a	AB698867a	AB828564f
*Orobdella octonaria* Oka, 1895	KUZ Z181 Topotype	AB698870e	AB698871a	AB679708b	AB679709b	AB828562f
*Orobdella shimadae* Nakano, 2011b	KUZ Z128 Holotype	AB663663e	AB698875a	AB679676b	AB679677b	AB828557f
*Orobdella tsushimensis* Nakano, 2011a	KUZ Z134 Holotype	AB663653e	AB698872a	AB679662b	AB679663b	AB828554f
*Orobdella whitmani* Oka, 1895	KUZ Z45 Topotype	AB663657e	AB698874a	AB679668b	AB679669b	AB828556c
*Erpobdella japonica* Pawłowski, 1962	KUZ Z178	AB663648e	AB698879a	AB679654b	AB679655b	AB828542f
*Gastrostomobdella monticola* Moore, 1929	UNIMAS/A3/BH01/10	AB663649e	AB698880a	AB679656b	AB679657b	AB828543f
*Mimobdella japonica* Blanchard, 1897	KUZ Z179	AB663650e	AB698881a	AB679658b	AB679659b	AB828544f
*Odontobdella blanchardi* (Oka, 1910)	KUZ Z180	AB663651e	AB938012	AB938004	AB937995	AB938014

Sources: a Nakano (2012a); b Nakano (2012b); c Nakano and Gongalsky (2014); d Nakano and Lai (2012); e Nakano et al. (2012); f Nakano and Seo (2014).

### Molecular phylogenetic and genetic distance analyses

Sixty-six previously published sequences (Nakano 2012a, b, Nakano and Gongalsky 2014, Nakano and Lai 2012, Nakano et al. 2012, Nakano and Seo 2014) were obtained from the INSDC and used for the molecular phylogenetic analyses (Table 2). Four erpobdelliform species, *Erpobdella japonica* Pawłowski, 1962, *Gastrostomobdella monticola* Moore, 1929, *Mimobdella japonica* Blanchard, 1897, and *Odontobdella blanchardi* (Oka, 1910), were used as outgroup taxa.

The phylogenetic position of the new species within the genus *Orobdella* was estimated based on sequences of nuclear 18S and H3 and mitochondrial COI, tRNA^Cys^–16S, and ND1. Sequences of nuclear H3 and mitochondrial COI were aligned by eye because there were no indels. Nuclear 18S and mitochondrial tRNA^Cys^–16S and tRNA^Leu^–ND1 were aligned using MATTF L-INS-I (Katoh et al. 2005). Then, the tRNA^Leu^ region was removed from each sequence of tRNA^Leu^–ND1. The length of the aligned 18S sequences was 1845 bp, that of H3 was 327 bp, that of COI was 1266 bp, that of tRNA^Cys^–16S was 1107 bp, and that of ND1 was 633 bp. The concatenated sequences thus yielded 5,124 bp positions.

Phylogenetic trees were constructed using maximum likelihood (ML) and Bayesian inference (BI) models. ML phylogenies were calculated using TREEFINDER v. October 2008 (Jobb et al. 2004) with the PHYLOGEARS v. 2.0 tool package (Tanabe 2008), followed by nonparametric bootstrapping (BS) (Felsenstein 1985) conducted with 1,000 replicates. The best-fit models for each partition were selected based on the Akaike Information Criterion (Akaike 1974) using KAKUSAN4 (Tanabe 2011): for 18S, TN93 with gamma distribution (+G) and proportion of invariant sites (+I); for the first, second, and third positions of H3, respectively, a homogenous (+H) TN93 model, JC69+H, and J2+G; for the first, second, and third positions of COI, respectively, TN93+G+I, TVM+I, and TIM+G; for tRNA^Cys^–16S, GTR+G; and for the first, second, and third positions of ND1, respectively, GTR+G+I, HYK85+G, and J2+G. BI and Bayesian posterior probabilities (BPPs) were estimated using MRBAYES v. 3.2 (Ronquist et al. 2012). The best-fit models for each partition were identified with the Bayesian information criterion (Schwarz 1978) using KAKUSAN4: for 18S, K80+G; for the first, second and third positions of H3, respectively, JC69+H, JC69+H, and HKY+G; for the first, second, and third positions of COI, respectively, GTR+G+I, F81+I, and HKY+G; for tRNA^Cys^–16S, GTR+G; and for the first, second, and third positions of ND1, respectively, GTR+G, HKY85+G, and HKY85+G. Two independent runs of four Markov chains were conducted for 10 million generations, and the tree was sampled every 100 generations. The parameter estimates and convergence were checked using TRACER v. 1.5 (Rambaut and Drummond 2009), and the first 25,001 trees were discarded based on these results.

The phylogenetic relationships of the specimens of the new species were reconstructed based on sequences of mitochondrial regions. The alignment of the sequences as well as the reconstruction of the ML and BI phylogenies was accomplished followed the methods described above. The length of the aligned COI was 1266 bp, that of tRNA^Cys^–16S was 1056 bp, and that of ND1 was 579 bp. Thus, the concatenated sequences yielded 2,901 bp positions. The best-fit models for each partition selected for the ML phylogenies were as follows: for the first, second, and third positions of COI, respectively, TN93+G, TVM+H, and TN93+G; for tRNA^Cys^–16S, GTR+G; and for the first, second, and third positions of ND1, respectively, TN93+G, HKY85+H, and HYK85+G. The best-fit models identified for each partition for the BI analyses were as follows: the first, second, and third positions of COI, respectively, GTR+G, F81+H, and HKY85+G; for tRNA^Cys^–16S, GTR+G; and for the first, second, and third positions of ND1, respectively, GTR+G, F81+H, and HKY85+G. For BI and BPPs, two independent runs of four Markov chains were conducted for 6 million generations, and the tree was sampled every 100 generations. The first 15,001 trees were eliminated based on the results of the parameter estimates and convergence.

Nodes with BS values higher than 70% were considered sufficiently resolved (Hillis and Bull 1993). Nodes with BPPs higher than 95% were considered statistically significant (Leaché and Reeder 2002).

Pairwise comparisons of the Kimura-2-parameter (K2P) distance (Kimura 1980) for the COI sequences (1266 bp) obtained from the specimens of the new species were calculated using MEGA5 (Tamura et al. 2011).

## Taxonomy

### Family Orobdellidae

Taxon classificationAnimaliaArhynchobdellidaOrobdellidae

Nakano, Ramlah & Hikida, 2012

http://zoobank.org/5F5BABE8-BD26-4FC7-9593-F73E62E26122

### Genus Orobdella

Taxon classificationAnimaliaArhynchobdellidaOrobdellidae

Oka, 1895

http://zoobank.org/FA8333ED-8C17-41FD-AFC1-62A4F98D4AC1

### Orobdella masaakikuroiwai sp. n.

Taxon classificationAnimaliaArhynchobdellidaOrobdellidae

http://zoobank.org/72F9627C-763A-49D9-9C97-E15B2FD856AA

Figs 2 , 3 , 4 , 5

#### Diagnosis.

Body length of mature individual less than 4 cm. Somite IV uniannulate, somites VIII–XXV quadrannulate. Clitellum in XI b5 to XIII a2. Pharynx reaching to XIV. Gastropore conspicuous in middle of XIII a1. Gastroporal duct bulbous, winding at junction with gastropore. Male gonopore in middle of XI b6, female gonopore inconspicuous in middle of XIII a1, behind gastropore, gonopores separated by 1/2 + 4 + 1/2 annuli. Paired epididymides in XV/XVI–XVI b5/b6 to XVII b5/b6–XVIII/XIX, occupying 7–10 annuli (i.e. one and a half to two and a half somites). Atrial cornua developed, ovate.

#### Type materials (see Fig. 1 for the locality number).

**Holotype.** KUZ Z694, holotype, dissected, collected from under a rock along a forest road at Mt. Mitsugaisan, Ina, Nagano Pref., Japan (35°47.72'N, 138°04.70'E; Alt. 875 m; locality number 4), by TN on 20 July 2012.

**Paratypes.** Four paratypes from the type locality by TN on 20 July 2012: KUZ Z690, Z691 (35°47.72'N, 138°04.69'E; Alt: 872 m), and KUZ Z692, Z693 (35°47.74'N, 138°04.69'E; Alt: 872 m). KUZ Z693, dissected.

#### Additional materials (see Fig. 1 and Table 1 for the locality numbers).

In total, 11 specimens examined. KUZ Z684–Z686 (three specimens), collected from under rocks in Akiruno (locality number 1), by TN: KUZ Z684, from along a mountain trail at Mt. Kariyoseyama (35°42.37'N, 139°12.03'E; Alt. 341 m) on 29 March 2010; KUZ Z685, from along Ohikagedori Road (35°43.33'N, 139°11.98'E; Alt. 230 m) on 30 March 2010; KUZ Z686, from along Bonborisen Forest Road (35°47.73'N, 139°11.01'E; Alt. 284 m) on 30 March 2010. KUZ Z687, Z688 (two specimens), from under rocks along a forest road in Namesawakeikoku Valley (locality number 2), by TN on 9 July 2011: KUZ Z687 (34°50.59'N, 138°54.69'E; Alt. 551 m); KUZ Z688 (34°50.50'N, 138°54.59'E; Alt. 576 m). KUZ Z689, from under fallen leaves along a forest road at Shirabisotoge Pass (35°26'N, 138°01'E; Alt. 1840 m; locality number 5), by Yoshiko Yamane on 14 October 2011. KUZ Z695, Z696 (two specimens), from under rocks in Shiojidaira Nature Park (locality no 7), by TN on 10 August 2012: KUZ Z695 (35°40.62'N, 137°50.48'E; Alt. 1304 m); KUZ Z696 (35°40.66'N, 137°50.48'E; Alt. 1315 m). KUZ Z697, Z698 (two specimens), from under rocks along a mountain stream in Ikuta (locality no 6), by TN on 10 August 2012: KUZ Z697 (35°33.67'N, 138°00.04'E; Alt. 1098 m); KUZ Z698 (35°33.68'N, 138°00.04'E; Alt. 1099 m). KUZ Z699, from under fallen leaves near Shibunoyu (36°02.1'N, 138°19.5'E; Alt. 1860 m; locality number 3), by Yume Imada on 6 October 2012. KUZ Z684, Z687, Z689, Z696, Z697 and Z699 (six specimens), dissected.

#### Etymology.

The specific name is a noun in the genitive case formed directly from the name of Mr Masaaki Kuroiwa, who generously accompanied the field survey in Nagano Prefecture.

#### Description of holotype.

**Figure 2.**
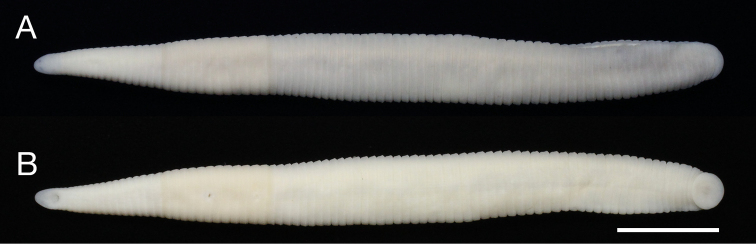
*Orobdella masaakikuroiwai* sp. n., holotype, KUZ Z694. **A** Dorsal and **B** ventral views. Scale bar, 5 mm.

**Figure 3.**
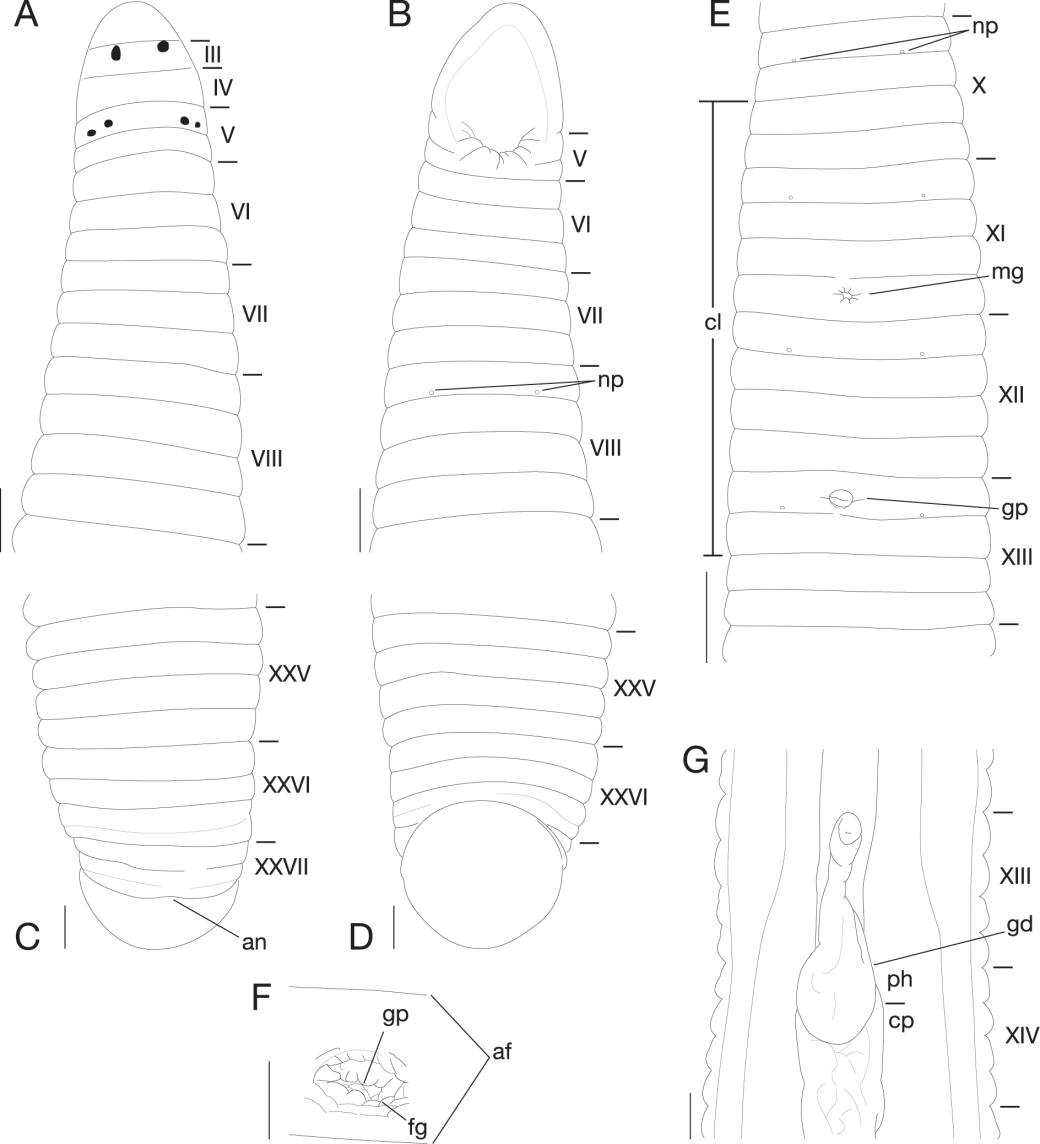
*Orobdella masaakikuroiwai* sp. n., holotype, KUZ Z694. **A** Dorsal and **B** ventral views of somites I–VIII. **C** Dorsal and **D** ventral views of somites XXV–XXVII and caudal sucker. **E** Ventral view of somites X–XIII. **F** Ventral view of gastropore and female gonopore. G Ventral view of gastroporal duct. Scale bars, 1 mm (**E**), 0.5 mm (**A**–**D**, **G**) and 0.25 mm (**F**). Abbreviations: af, annular furrow; an, anus; cl, clitellum; cp, crop; fg, female gonopore; gd, gastroporal duct; gp, gastropore; mg, male gonopore; np, nephridiopore; and ph, pharynx.

Body firm and muscular, elongate, with constant width in caudal direction, dorsoventrally compressed, BL 34.0 mm, BW 3.42 mm (Fig. 2). Caudal sucker ventral, elliptic, CL 1.7 mm (minor axis), CW 1.9 mm (major axis) (Figs 2B, 3D).

Somite I completely merged with prostomium (Fig. 3A). Somites II–IV uniannulate, II not separated from I (Fig. 3A). Somite V biannulate, (a1 + a2) = a3; a3 forming posterior margin of oral sucker (Fig. 3A, B). Somites VI, VII triannulate, a1 = a2 = a3 (Fig. 3A, B). Somites VIII–XXV quadrannulate, a1 = a2 = b5 = b6 (Fig. 3A–E); b5 of X and a2 of XIII respectively being first and last annuli of clitellum (Fig. 3E). Somite XXVI triannulate, with slight furrow in a3, a1 > a2 < a3 (b5 = b6); a3 being ventrally last complete annuls (Fig. 3C, D). Somite XXVII biannulate, with slight dorsal furrow in last annulus; anus behind it with no post-anal annulus (Fig. 3C).

**Figure 4.**
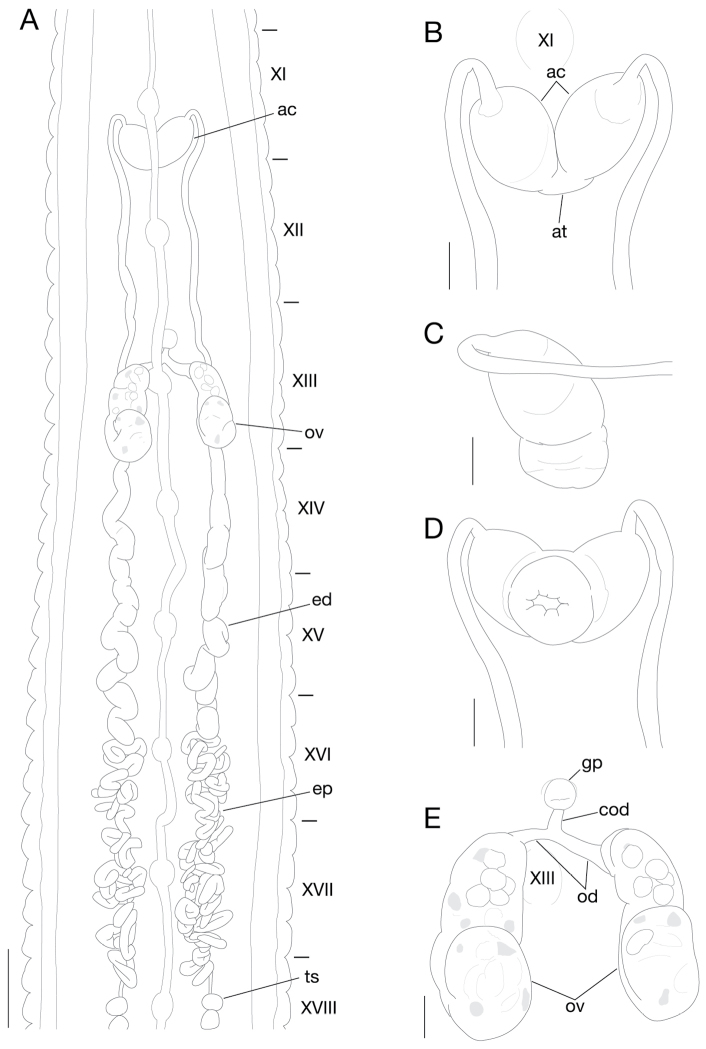
*Orobdella masaakikuroiwai* sp. n., holotype, KUZ Z694. **A** Dorsal view of reproductive system including ventral nervous system. **B** Dorsal, **C** lateral, and **D** ventral views of male atrium: **B** including position of ganglion XI. **E** Dorsal view of female reproductive system including position of ganglion III. Scale bars, 1 mm (**A**) and 0.25 mm (**B**–**E**). Abbreviations: ac, atrial cornua; at, atrium; cod, common oviduct; ed, ejaculatory duct; ep, epididymis; gp, gastropore; od, oviduct; ov, ovisac; and ts, testisacs.

Anterior ganglionic mass in VI a2 and a3. Ganglia VII–X, of each somite, in a2 (Fig. 4A). Ganglion XI in a2 and b5 (Fig. 4A). Ganglia XII–XVIII, of each somite, in a2 (Fig. 4A). Ganglia XIX, XX, of each somite, in a1 and a2. Ganglia XXI, XXII, of each somite, in a2. Ganglion XXIII in a1 and a2. Ganglion XXIV in a1. Ganglion XXV in XXIV b6 and XXV a1. Ganglion XXVI in b5 and b6 of XXV. Posterior ganglionic mass in a1–a3 of XXVI.

Eyes in three pairs, first pair dorsally on anterior margin of III, second and third pairs dorsolaterally on posterior margin of V (a1 + a2) (Fig. 3A). Nephridiopores in 17 pairs, one each situated ventrally at posterior margin of a1 of each somite in VIII–XXIV (Fig. 3B, E). Papillae numerous, minute, hardly visible, one row on every annulus.

Pharynx agnathous, euthylaematous, reaching to XIV a1/a2 (Fig. 3G). Crop tubular, reaching to XIX b5/b6 (Fig. 3G). Gastropore conspicuous, ventral in middle of XIII a1 (Fig. 3E, F). Gastroporal duct bulbous, slightly winding at junction with gastropore, joining with crop in XIV b5 (Fig. 3G). Intestine tubular, acecate, reaching to XXIV a1/a2. Rectum tubular, thin-walled, descending to anus.

Male gonopore in middle of XI b6 (Fig. 3E). Female gonopore in middle of XIII a1, inconspicuous, located posterior to gastropore (Fig. 3G). Gonopores separated by 1/2 + 4 + 1/2 annuli (Fig. 3E). Testisacs multiple, one or two on each side in each annulus, in XVIII a2 to XXV a1 (Fig. 4A). Paired epididymides in XVI a2 to XVIII a1, occupying 8 annuli (Fig. 4A). Ejaculatory bulbs absent. Paired ejaculatory ducts in XI a2/b5 to XVI a2, coiled in position posterior to ovisacs; each duct crossing ventrally beneath each ovisac, then loosely curved in position anterior to ovisacs; each widening from respective junction with epididymis, narrowing at junction with atrial cornua, then turning sharply inward toward atrial cornua with pre-atrial loop reaching to anterior margin of XI b5 (Fig. 4A). Pair of muscular atrial cornua ovate, in XI b5 and b6 (Fig. 4A–D). Atrium short, muscular, globular in XI b6 (Fig. 4B–D). Penis sheath and penis absent. Paired ovisacs elongated globular, one each in XIII a2–b6 (Fig. 4A, E). Oviducts thin-walled, left oviduct crossing ventrally beneath nerve cord; both oviducts converging into common oviduct in XIII a1/a2 (Fig. 4A, E). Common oviduct thin-walled, short, directly descending to female gonopore (Fig. 4E).

#### Variation.

BL 22.4 (KUZ Z686) –35.2 (KUZ Z684) mm, BW 2.3 (KUZ Z691) –3.5 (KUZ Z684) mm, CL 1.1 (KUZ Z686)–1.7 (KUZ Z693) mm, CW 1.1 (KUZ Z686)–2.1 (KUZ Z689) mm. Somites III, IV uniannulate, each with slight dorsal furrow (KUZ Z695). Somite XXVI variable; often dorsally quadrannulate, ventrally triannulate, rarely with slight ventral furrow in a3; KUZ Z699 with quadrannulate; KUZ Z698, Z691 with triannulate with slight dorsal furrow in a3; KUZ Z689 with triannulate. Somite XXVII biannulate, or uniannulate with slight dorsal furrow. Eyes in three pairs; KUZ Z699 with one eye dorsoleft on posterior margin of III. Pharynx reaching to XIII/XIV–XIV a2/b5. Crop reaching to XIX b5/b6–XX a1. Gastropore occasionally slightly posterior to middle of XIII a1. Gastroporal duct joining with crop in XIV a1/a2–XIV b6; KUZ Z687 with thick, tubular duct. Intestine reaching to XXIII/XXIV–XXV a2. Male gonopore rarely slightly anterior to middle of XI b6, or slightly posterior to middle of XI b6. Female gonopore occasionally slightly posterior to middle of XIII a1. Testisacs in XVII b6–XIX a1 to XXIV b5–XXV a2. Epididymides in XV/XVI–XVI b5/b6 to XVII b5/b6–XVIII/XIX; occupying 7–10 annuli. Atrial cornua generally ovate; KUZ Z696 ellipsoid; KUZ Z687 fusiform. Pre-atrial loop absent, or reaching to middle of XI b5 (KUZ Z693, Z697). Ovisacs often in XIII a2–b6; KUZ Z687, Z699 in XIII a2, b5; KUZ Z696 right one in XIII a2–XIV a1/a2, left one in XIII a2–XIV a1. Right or left oviduct crossing ventrally beneath nerve cord; KUZ Z684, Z693 both oviducts converging into common oviduct in XIII a2.

#### Coloration.

**Figure 5.**
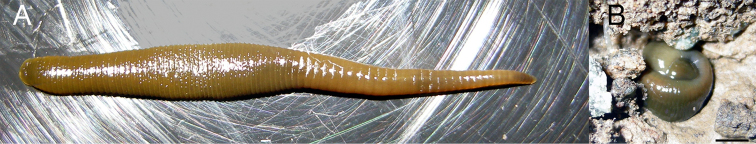
*Orobdella masaakikuroiwai* sp. n., paratype, KUZ Z690. **A** Dorsal view of live animal. **B** Live animal found curled up under a stone at the type locality: scale bar, 2 mm.

In life, dorsal surface ochre (Fig. 5), whitish brown, or brown, ventral surface grayish white or yellowish white; individuals from Shizuoka Pref. (KUZ Z687, Z688), dorsal surface whitish yellow. Colour faded in preservative, rarely with one dorsal black line from VII a3–IX a2 to XIX b5–XXVI b6 (KUZ Z691, Z693, Z694, Z698).

#### Distribution (see Fig. 1 for the locality numbers).

This species was primarily collected from localities in Nagano Prefecture: the east-central part (locality number 3), and the southeastern part along the Inadani Basin (locality numbers 4–7). This species was also found in the western mountainous part of the Metropolitan Tokyo area (locality number 1), as well as in the Amagi Mountain Range in the central part of the Izu Peninsula, Shizuoka Prefecture (locality number 2). The locality data for this species suggested that *Orobdella masaakikuroiwai* sp. n. would be widely distributed in mountainous regions such as the southwestern part of the Kanto Region and the southeastern part of the Chubu Region, Honshu, Japan. The lowest elevation among the localities was 230 m above sea level (a.s.l.) (locality number 1), and the highest was ca. 1860 m a.s.l. (locality number 3).

#### Natural history.

This species was generally found curled up under rocks or fallen leaves in moist mountainous habitats (Fig. 5B). Soil was sometimes observed in the digestive tract during specimen dissection. This species is therefore considered an earthworm-feeder as are the other known *Orobdella* leeches.

Mature leeches with an obvious clitellum were collected on 20 July (KUZ Z690, Z691, Z693, Z694) and 10 August (KUZ Z697) at two sites in Nagano Prefecture (locality numbers 4 and 7, elevation ca. 875 m and 1098 m, respectively). These findings indicate that the reproductive season of this species may begin in mid-to-late July.

#### Remarks.

Although the leech specimens examined in this study were small (up to 35 mm), several individuals, including the holotype, were determined to be mature due to the possession of an obvious clitellum and developed testisacs. Specimen KUZ Z687 possessed a tubular gastroporal duct and fusiform atrial cornua. Immature leeches may have these characteristics, because the sperm ducts and testisacs of specimen KUZ Z687 are undeveloped and barely detectable.

The new species unambiguously belongs to the genus *Orobdella* as it has all the generic diagnostic characteristics (see Nakano et al. (2012) for the generic diagnosis): post-anal annulus absent; pharynx agnathous, euthylaematous; gastropore in XIII; gastroporal duct lying on female organ; gonopores separated by more than one full somite; testisacs multiple; male atrium in XI without penis sheath and penis; ovisacs globular in XIII; female median reproductive system essentially lacking.

According to previous taxonomic studies (Nakano 2010, 2011a, 2012b, Nakano and Gongalsky 2014, Nakano and Lai 2012, Nakano and Seo 2012, 2014), *Orobdella masaakikuroiwai* sp. n. differs from the six other quadrannulate species (i.e., *Orobdella esulcata* Nakano, 2010, *Orobdella kawakatsuorum*, *Orobdella ketagalan* Nakano & Lai, 2012, *Orobdella koikei*, *Orobdella tsushimensis* Nakano, 2011a, and *Orobdella whitmani* Oka, 1895) by the following combination of characteristics (Table 3): body length less than 4 cm, IV uniannulate, gonopores separated by 1/2 + 4 + 1/2, XXV quadrannulate, gastroporal duct bulbous, epididymides in XVI to XVIII, atrial cornua ovate. Among the six above-listed quadrannulate species, only *Orobdella whitmani* is present in Honshu. Both *Orobdella masaakikuroiwai* sp. n. and *Orobdella whitmani* possess 1/2 + 4 + 1/2 annuli between the gonopores, a bulbous gastroporal duct, and epididymides in XVI–XVIII. Thus, it is difficult to distinguish these two species using these diagnostic features. However, *Orobdella whitmani* is a large species and grows up to ca. 10 cm (Nakano 2010, Oka 1895). Therefore, *Orobdella masaakikuroiwai* sp. n. clearly differs from mature individuals of *Orobdella whitmani* in body length. However, distinguishing the new species from a small juvenile of *Orobdella whitmani* can be complex. Because immature individuals of *Orobdella whitmani* possess a tubular gastroporal duct (Nakano, unpublished observation) and mature individuals of *Orobdella masaakikuroiwai* sp. n. possess a bulbous gastroporal duct, the characteristics of the duct could be used to distinguish between the two. However, insofar as immature leeches of both species have a tubular gastroporal duct, this characteristic is not useful for discriminating between immature individuals of *Orobdella masaakikuroiwai* sp. n. and *Orobdella whitmani*. DNA data might be useful for identification, similar to the DNA barcoding of freshwater leeches (e.g. Oceguera-Figueroa et al. (2010)). In addition to DNA data, interbreeding experiments or karyological studies may be crucial for definitive clarification between *Orobdella masaakikuroiwai* sp. n. and *Orobdella whitmani* as is the case with the species of *Hirudo* Linnaeus, 1758 in Europe (Petrauskiene et al. 2009, Utevsky et al. 2009).

**Table 3.** Comparison of morphological characters between *Orobdella masaakikuroiwai* sp. n. and six quadrannulate congeneric species.

**Character**	*Orobdella masaakikuroiwai* sp. n.	*Orobdella esulcata* Nakano 2010	*Orobdella kawakatsuorum* Richardson, 1975	*Orobdella ketagalan* Nakano & Lai, 2012	*Orobdella koikei* Nakano, 2012b	*Orobdella tsushimensis* Nakano, 2011a	*Orobdella whitmani* Oka, 1895
**Body length of mature individual**	less than 4 cm	up to ca 10 cm	up to ca 10 cm	up to ca 10 cm	less than 4 cm	up to ca 10 cm	up to ca 10 cm
**Annulation of IV**	uniannulate	uniannulate	biannulate	uniannulate	uniannulate	uniannulate	uni- or biannulate
**Number of annuli between gonopores**	1/2 + 4 + 1/2	2/3 + 4 + 1/3	6	1/2 + 4 + 1/2	1/2 + 4 + 1/2	1/2 + 5	1/2 + 4 +1/2
**Annulation of XXV**	quadrannulate	quadrannulate	quadrannulate	quadrannulate	triannulate	quadrannulate	quadrannulate
**Gastroporal duct**	bulbous	tubular, but bulbous at junction with gastropore	simple tubular	simple tubular	bulbous	bulbous	bulbous
**Epididymides**	XVI to XVIII	XVI to XX	XVI to XVII	absent	XV to XX	XVII to XIX	XVI to XVIII
**Atrial cornua**	ovate	ovate	undeveloped	undeveloped	ovate	ovate	ovate

The quadrannulate *Orobdella masaakikuroiwai* sp. n. is unequivocally distinguishable from the four species *Orobdella dolichopharynx* Nakano, 2011b, *Orobdella ijimai* Oka, 1895, *Orobdella mononoke* Nakano, 2012a and *Orobdella shimadae* Nakano, 2011b, due to their sexannulate mid-body somites, as well as *Orobdella octonaria*, which possesses octannulate mid-body somites.

### Molecular phylogenies and genetic distances

**Figure 6.**
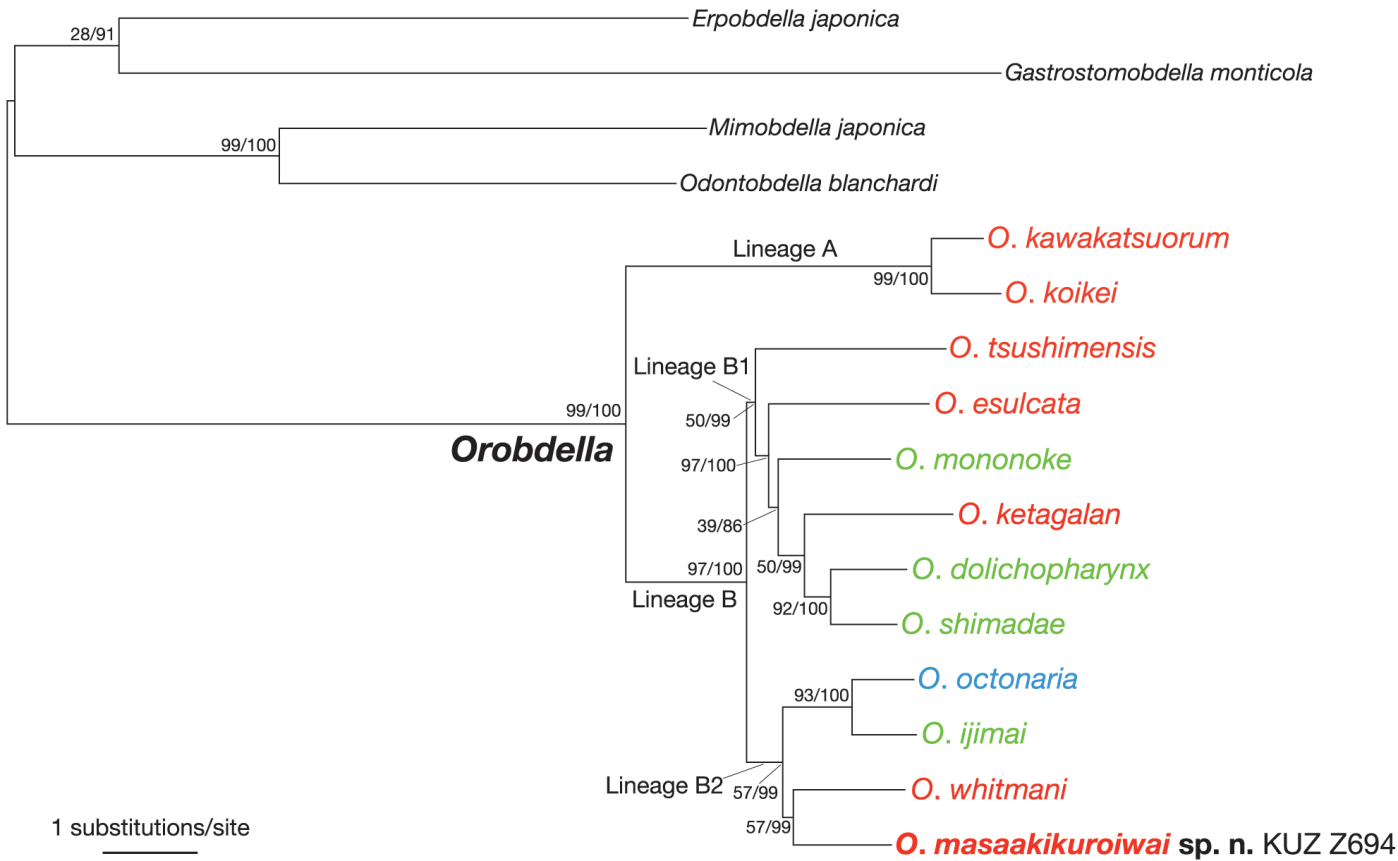
The ML tree (ln *L* = −23350.60) for 5,124 bp of nuclear 18S rDNA and histone H3, and mitochondrial COI, tRNACys, tRNAMet, 12S rDNA, tRNAVal, 16S rDNA, and ND1 markers. A species name of *Orobdella* in red indicates a quadrannulate species; in green, sexannulate; and in blue, octannulate. The numbers associated with the nodes represent the bootstrap values for ML (BS)/and Bayesian posterior probabilities (BPPs).

The ML tree (ln *L* = −23350.60) (Fig. 6) for estimating the phylogenetic position of the new species had an identical topology to the BI tree (not shown). The monophyly of the genus *Orobdella* was confirmed (BS = 99%, BPP = 100%) The genus was divided into two lineages (hereafter lineages A and B). Lineage A consisted of *Orobdella kawakatsuorum* and *Orobdella koikei* (BS = 99%, BPP = 100%). Monophyletic lineage B (BS = 97%, BPP = 100%) included the remaining 10 species (including the new species), and was divided into two sub-lineages (hereafter lineages B1 and B2). The monophyly of lineage B1, which consisted of six species, was not well supported by the ML analysis (BS = 50%, BPP = 99%). Lineage B2 included four species, but the monophyly of this lineage was also not well supported by the ML analysis (BS = 57%, BPP = 99%). The new species, *Orobdella masaakikuroiwai* sp. n., was part of lineage B2, and was a sister taxon of *Orobdella whitmani* within this lineage. However, this relationship was not fully supported by the ML analysis (BS = 57%, BPP = 99%).

**Figure 7.**
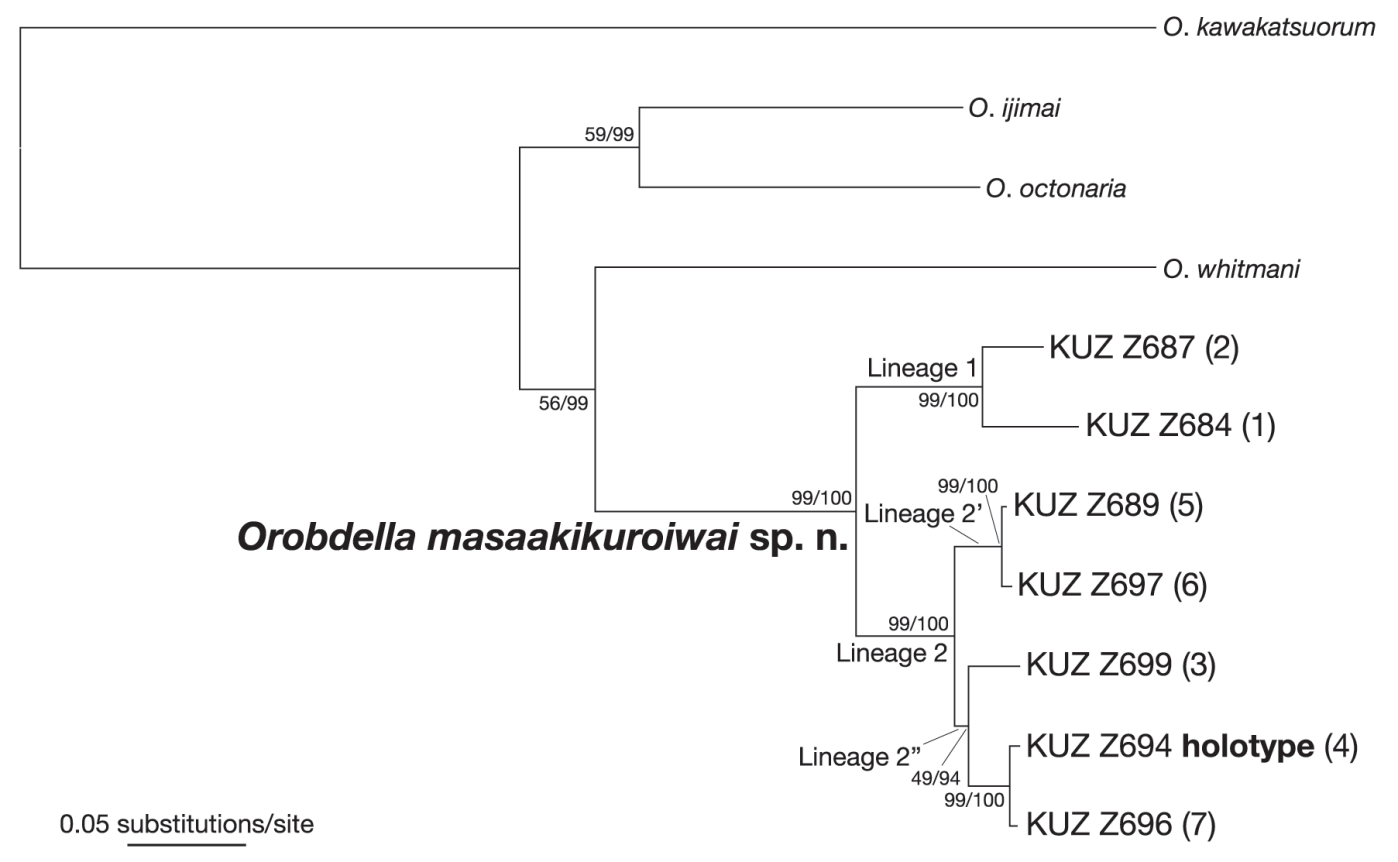
The ML tree (ln *L* = −8756.30) for 2,901 bp of mitochondrial COI, tRNACys, tRNAMet, 12S rDNA, tRNAVal, 16S rDNA, and ND1 markers. Voucher numbers of the specimens of *Orobdella masaakikuroiwai* sp. n. are accompanied by the collection locality numbers (see Fig. 1). The numbers associated with the nodes represent the bootstrap values for ML (BS)/and Bayesian posterior probabilities (BPPs).

The ML tree (ln *L* = −8756.30) (Fig. 7) for reconstructing the phylogenetic relationships of the new species had an identical topology to the BI tree (not shown). The monophyly of the specimens identified as *Orobdella masaakikuroiwai* sp. n. was well supported (BS = 99%, BPP = 100%). This clade was divided into two subclades (hereafter lineages 1 and 2). Monophyletic lineage 1 (BS = 99%, BPP = 100%) consisted of two specimens, KUZ Z684 (locality number 1; Tokyo Metropolis), and Z687 (locality number 2; Shizuoka Prefecture). The monophyly of lineage 2 was well supported (BS = 99%, BPP = 100%). Lineage 2 contained five specimens from Nagano Prefecture including the holotype, and consisted of two subclades (hereafter lineages 2’ and 2”). The monophyly of lineage 2’ was well supported (BS = 99%, BPP = 100%). This lineage included two specimens, KUZ Z689 (locality number 5) and Z697 (locality number 6), observed in the southern part of the prefecture. The monophyly of lineage 2” was not well supported in the ML analysis (BS = 49%, BPP = 94%). Lineage 2” contained three specimens, KUZ Z694 (holotype; locality number 4), Z696 (locality number 7), and Z699 (locality number 3) collected from the east-central and mid-southern parts of Nagano Prefecture. KUZ Z694 and Z696 formed a monophyletic lineage (BS = 99%, BPP = 100%) within lineage 2”.

The COI K2P distance within *Orobdella masaakikuroiwai* sp. n. was 0.5–6.7% (mean = 4.4%) (Table 4). The genetic divergence between lineages 1 and 2 was 5.8–6.7% (mean = 6.3%), and that between lineages 2’ and 2’’ was 2.7–3.5% (mean = 3.2%). The COI K2P distance between *Orobdella masaakikuroiwai* sp. n. and *Orobdella whitmani* (KUZ Z45, topotype) was 10.4–11.7% (mean = 11.0%).

**Table 4.** Kimura-2-parameter distances for the 1266 bp for the COI sequences of *Orobdella masaakikuroiwai* sp. n. specimens, with associated collection locality numbers (see Fig. 1 and Table 1).

Specimen (locality number)	1	2	3	4	5	6	7
1: KUZ Z684 (1)							
2: KUZ Z687 (2)	0.046						
3: KUZ Z689 (5)	0.065	0.061					
4: KUZ Z694 (4)	0.065	0.058	0.033				
5: KUZ Z696 (7)	0.063	0.059	0.035	0.005			
6: KUZ Z697 (6)	0.066	0.067	0.006	0.034	0.034		
7: KUZ Z699 (3)	0.063	0.059	0.027	0.023	0.023	0.027	

## Discussion

The current molecular phylogenies showed that the specimens morphologically identified as the new species form a monophyletic group with strong support values. In addition, the K2P genetic distance of the COI sequences detected within the specimens was 0.5–6.7% (mean = 4.4%). Nakano (2012b) stated that the COI K2P distance between the sister species of *Orobdella*, *Orobdella kawakatsuorum* and *Orobdella koikei*, was 8.1–9.9% (mean = 9.0%). Therefore, the present genetic analyses support the taxonomic designation of the specimens examined in this study as belonging to the new species, *Orobdella masaakikuroiwai* sp. n.

*Orobdella masaakikuroiwai* sp. n. was divided into two lineages (lineages 1 and 2) according to the molecular phylogenetic analyses. Lineage 1 consists of the individuals inhabiting the Kanto Region (KUZ Z684, locality number 1) and the Izu Peninsula (KUZ Z687, locality number 2). The Izu Peninsula is located on the Philippine Sea Plate and collided with Honshu island around 1 million years ago (Kitazato 1997). Therefore, *Orobdella masaakikuroiwai* sp. n. likely migrated into the peninsula after this collision event. In addition to lineages 1 and 2 composed of specimens from the mountainous region of Nagano Prefecture, the individuals of *Orobdella masaakikuroiwai* sp. n. were sub-divided into central (lineage 2’’; locality numbers 3, 4, 7) and southern (lineage 2’; locality numbers 5, 6) phylogroups. The Ina Basin is located in the southern part of Nagano Prefecture along the Tenryu River. Mountain districts are present to the east (including locality numbers 4–6) and west (containing locality number 7) along this basin. The specimen from Shiojidaira is the closest to the holotype from Mt. Mitsugaisan even though the Ina Basin separates the mountainous regions. In addition, the COI divergence between the two specimens from Shiojidaira (KUZ Z696) and Mt. Mitsugaisan (KUZ Z694) was low (0.5%). This may indicate that *Orobdella masaakikuroiwai* sp. n. leeches in this area have recently dispersed. The same low genetic distance (0.6%) was detected between the specimens collected from the southern part of Nagano Prefecture (KUZ Z689, locality number 5, and KUZ Z697, locality number 6).

*Orobdella masaakikuroiwai* sp. n. is the second known species in which the body length of a mature individual is less than 4 cm. *Orobdella masaakikuroiwai* sp. n. is syntopic with *Orobdella octonaria* in the Izu Peninsula (locality number 2), and the distribution of this new species partly overlaps with that of the latter species (Nakano, unpublished data). In addition, both *Orobdella koikei* and *Orobdella kawakatsuorum* are present in Hokkaido (Nakano 2012b). Therefore, a difference in the body size of mature individuals may allow different species of *Orobdella* to coexist in the same region. The phylogeny indicates that the small size of mature leeches likely evolved in parallel within *Orobdella*. *Orobdella whitmani* is the sister species of *Orobdella masaakikuroiwai* sp. n. and grows to ca. 10 cm. In addition, *Orobdella ijimai* and *Orobdella octonaria* are close congeners of *Orobdella masaakikuroiwai* sp. n. and *Orobdella whitmani*, and they grow to ca. 10 cm and ca. 20 cm, respectively. Therefore, the intermediate size of mature individuals may be a plesiomorphic characteristic of the clade consisting of these four species. However, several undescribed species of *Orobdella* are known including small-sized species (Nakano, unpublished observation). Further faunal and systematic studies will help to elucidate the evolutionary and biogeographical history of the predaceous genus *Orobdella*.

## Supplementary Material

XML Treatment for Orobdellidae

XML Treatment for Orobdella

XML Treatment for Orobdella masaakikuroiwai

## References

[B1] Akaike H (1974) A new look at the statistical model identification. IEEE Transactions on Automatic Control 19: 716–723

[B2] Apakupakul K Siddall ME Burreson EM (1999) Higher level relationships of leeches (Annelida: Clitellata: Euhirudinea) based on morphology and gene sequences. Molecular Phylogenetics and Evolution 12: 350–359. doi: 10.1006/mpev.1999.0639 10413628 10.1006/mpev.1999.0639

[B3] Blanchard R (1897) Hirudinées du Musée de Leyde. Notes from the Leyden Museum 19: 73–113

[B4] Colgan DJ McLauchlan A Wilson GDF Livingston SP Edgecombe GD Macaranas J Cassis G Gray MR (1998) Histone H3 and U2 snRNA DNA sequences and arthropod molecular evolution. Australian Journal of Zoology 46: 419–437. doi: 10.1071/ZO98048

[B5] Felsenstein J (1985) Confidence limits on phylogenies: an approach using the bootstrap. Evolution 39: 783–791. doi: 10.2307/2408678 10.1111/j.1558-5646.1985.tb00420.x 28561359

[B6] Folmer O Black M Hoeh W Lutz R Vrijenhoek R (1994) DNA primers for amplification of mitochondrial cytochrome c oxidase subunit I from diverse metazoan invertebrates. Molecular Marine Biology and Biotechnology 3: 294–299 7881515

[B7] Hillis DM Bull JJ (1993) An empirical test of bootstrapping as a method for assessing confidence in phylogenetic analysis. Systematic Biology 42: 182–192. doi: 10.1093/sysbio/42.2.182

[B8] Jobb G von Haeseler A Strimmer K (2004) TREEFINDER: a powerful graphical analysis environment for molecular phylogenetics. BMC Evolutionary Biology 4: . doi: 10.1186/1471-2148-4-18 10.1186/1471-2148-4-18 PMC459214 15222900

[B9] Katoh K Kuma K-i Toh H Miyata T (2005) MAFFT version 5: improvement in accuracy of multiple sequence alignment. Nucleic Acids Research 33: 511–518. doi: 10.1093/nar/gki198 15661851 10.1093/nar/gki198 PMC548345

[B10] Kimura M (1980) A simple method for estimating evolutionary rates of base substitutions through comparative studies of nucleotide sequences. Journal of Molecular Evolution 16: 111–120. doi: 10.1007/bf01731581 7463489 10.1007/BF01731581

[B11] Kitazato H (1997) Paleogeographic changes in central Honshu, Japan, during the late Cenozoic in relation to the collision of the Izu-Ogasawara Arc with the Honshu Arc. Island Arc 6: 144–157. doi: 10.1111/j.1440-1738.1997.tb00166.x

[B12] Leaché AD Reeder TW (2002) Molecular systematics of the eastern fence lizard (*Sceloporus undulatus*): a comparison of parsimony, likelihood, and Bayesian approaches. Systematic Biology 51: 44–68. doi: 10.1080/106351502753475871 11943092 10.1080/106351502753475871

[B13] Light JE Siddall ME (1999) Phylogeny of the leech family Glossiphoniidae based on mitochondrial gene sequences and morphological data. The Journal of Parasitology 85: 815–823 10577715

[B14] Linnaeus C (1758) Systema Naturae per Regna Tria Naturae, Secundum Classes, Ordines, Genera, Species, cum Characteribus, Diferentiis, Stinontmis, Locis. Salvius, Stockholm, 824 pp

[B15] Moore JP (1927) The segmentation (metamerism and annulation) of the Hirudinea. In: Harding WA Moore JP (Eds) The Fauna of British India, including Ceylon and Burma Hirudinea. Taylor & Francis, London, 1–12

[B16] Moore JP (1929) Leeches from Borneo with descriptions of new species. Proceedings of the Academy of Natural Sciences of Philadelphia 81: 267–295

[B17] Nakano T (2010) A new species of the genus *Orobdella* (Hirudinida: Arhynchobdellida: Gastrostomobdellidae) from Kumamoto, Japan, and a redescription of *O. whitmani* with the designation of the lectotype. Zoological Science 27: 880–887. doi: 10.2108/zsj.27.880 21039128 10.2108/zsj.27.880

[B18] Nakano T (2011a) A new species of *Orobdella* (Hirudinida: Arhynchobdellida: Gastrostomobdellidae) from Tsushima Island, Japan. Species Diversity 16: 39–47

[B19] Nakano T (2011b) Redescription of *Orobdella ijimai* (Hirudinida: Arhynchobdellida: Gastrostomobdellidae), and two new species of *Orobdella* from the Ryukyu Archipelago, Japan. Zootaxa 2998: 1–15

[B20] Nakano T (2012a) A new sexannulate species of *Orobdella* (Hirudinida, Arhynchobdellida, Orobdellidae) from Yakushima Island, Japan. ZooKeys 181: 79–93. doi: 10.3897/zookeys.181.2932 22539913 10.3897/zookeys.181.2932 PMC3332023

[B21] Nakano T (2012b) A new species of *Orobdella* (Hirudinida, Arhynchobdellida, Gastrostomobdellidae) and redescription of *O. kawakatsuorum* from Hokkaido, Japan with the phylogenetic position of the new species. ZooKeys 169: 9–30. doi: 10.3897/zookeys.169.2425 22371683 10.3897/zookeys.169.2425 PMC3278812

[B22] Nakano T (2012c) Redescription of *Orobdella octonaria* (Hirudinida: Arhynchobdellida: Orobdellidae) with designation of a lectotype. Species Diversity 17: 227–233. doi: 10.12782/sd.17.2.227

[B23] Nakano T Gongalsky KB (2014) First record of *Orobdella kawakatsuorum* (Hirudinida: Arhynchobdellida: Erpobdelliformes) from Kunashir Island, Kuril Islands. Biodiversity Data Journal 2: . doi: 10.3897/BDJ.2.e1058 10.3897/BDJ.2.e1058 PMC4030247 24855445

[B24] Nakano T Lai Y-T (2012) A new species of *Orobdella* (Hirudinida, Arhynchobdellida, Orobdellidae) from Taipei, Taiwan. ZooKeys 207: 49–63. doi: 10.3897/zookeys.207.3334 22855640 10.3897/zookeys.207.3334 PMC3409684

[B25] Nakano T Ramlah Z Hikida T (2012) Phylogenetic position of gastrostomobdellid leeches (Hirudinida, Arhynchobdellida, Erpobdelliformes) and a new family for the genus *Orobdella*. Zoologica Scripta 41: 177–185. doi: 10.1111/j.1463-6409.2011.00506.x

[B26] Nakano T Seo H-Y (2012) First record of *Orobdella tsushimensis* (Hirudinida: Arhynchobdellida: Orobdellidae) from Korea (Gageodo Island) and its molecular phylogenetic position within the genus. Species Diversity 17: 235–240. doi: 10.12782/sd.17.2.235

[B27] Nakano T Seo H-Y (2014) First record of *Orobdella tsushimensis* (Hirudinida: Arhynchobdellida: Gastrostomobdellidae) from the Korean Peninsula and molecular phylogenetic relationships of the specimens. Animal Systematics, Evolution and Diversity 30: 87–94. doi: 10.5635/ASED.2014.30.2.087

[B28] Oceguera-Figueroa A León-Régagnon V Siddall ME (2010) DNA barcoding reveals Mexican diversity within the freshwater leech genus *Helobdella* (Annelida: Glossiphoniidae). Mitochondrial DNA 21: 24–29. doi: 10.3109/19401736.2010.527965 21271855 10.3109/19401736.2010.527965

[B29] Oceguera-Figueroa A Phillips AJ Pacheco-Chaves B Reeves WK Siddall ME (2011) Phylogeny of macrophagous leeches (Hirudinea, Clitellata) based on molecular data and evaluation of the barcoding locus. Zoologica Scripta 40: 194–203. doi: 10.1111/j.1463-6409.2010.00465.x

[B30] Oka A (1895) On some new Japanese land leeches. (*Orobdella* nov. gen.). The Journal of the College of Science, Imperial University, Japan 8: 275–306

[B31] Oka A (1910) Key to Japanese leeches. Dobutsugaku Zasshi 22: 56–64

[B32] Pawłowski LK (1962) O występowaniu pijawki *Erpobdella octoculata* (L.) w Japonii. Zeszyty Naukowe Uniwersytetu Łódzkiego Nauki Matematiczno-przyrodnicze Seria II 12: 127–136

[B33] Petrauskiene L Utevska O Utevsky SY (2009) Can different species of medicinal leeches (*Hirudo* spp.) interbreed? Invertebrate Biology 128: 324–331. doi: 10.1111/j.1744-7410.2009.00180.x

[B34] Rambaut A Drummond AJ (2009) Tracer v. 1.5. http://tree.bio.ed.ac.uk/software/tracer/

[B35] Richardson LR (1971) Gastrostomobdellidae f. nov. and a new genus for the gastroporous *Orobdella octonaria* Oka, 1895, of Japan (Hirudinoidea: Arhynchobdellae). Bulletin of the National Science Museum (Tokyo) 14: 585–602

[B36] Richardson LR (1975) A new species of terricolous leeches in Japan (Gastrostomobdellidae, *Orobdella*). Bulletin of the National Science Museum Series A (Zoology) 1: 39–56

[B37] Ronquist F Teslenko M van der Mark P Ayres DL Darling A Höhna S Larget B Liu L Suchard MA Huelsenbeck JP (2012) MrBayes 3.2: Efficient Bayesian phylogenetic inference and model choice across a large model space. Systematic Biology 61: 539–542. doi: 10.1093/sysbio/sys029 22357727 10.1093/sysbio/sys029 PMC3329765

[B38] Sawyer RT (1986) Leech Biology and Behaviour. Clarendon Press, Oxford, 1065 pp

[B39] Schwarz G (1978) Estimating the dimension of a model. The Annals of Statistics 6: 461–464

[B40] Tamura K Peterson D Peterson N Stecher G Nei M Kumar S (2011) MEGA5: molecular evolutionary genetics analysis using maximum likelihood, evolutionary distance, and maximum parsimony methods. Molecular Biology and Evolution 28: 2731–2739. doi: 10.1093/molbev/msr121 21546353 10.1093/molbev/msr121 PMC3203626

[B41] Tanabe AS (2008) Phylogears v. 2.0. http://www.fifthdimension.jp

[B42] Tanabe AS (2011) Kakusan4 and Aminosan: two programs for comparing nonpartitioned, proportional and separate models for combined molecular phylogenetic analyses of multilocus sequence data. Molecular Ecology Resources 11: 914–921. doi: 10.1111/j.1755-0998.2011.03021.x 21592310 10.1111/j.1755-0998.2011.03021.x

[B43] Utevsky SY Kovalenko N Doroshenko K Petrauskiene L Klymenko V (2009) Chromosome numbers for three species of medicinal leeches (*Hirudo* spp.). Systematic Parasitology 74: 95–102. doi: 10.1007/s11230-009-9198-2 19731093 10.1007/s11230-009-9198-2

